# A high-quality chromosome-scale genome assembly of blood orange, an important pigmented sweet orange variety

**DOI:** 10.1038/s41597-024-03313-0

**Published:** 2024-05-06

**Authors:** Lei Yang, Honghong Deng, Min Wang, Shuang Li, Wu Wang, Haijian Yang, Changqing Pang, Qi Zhong, Yue Sun, Lin Hong

**Affiliations:** 1 Fruit Tree Research Institute, Chongqing Academy of Agricultural Sciences, Chongqing, 401329 China; 2 College of Horticulture, Fujian Agriculture and Forestry University, Fuzhou, 350002 China; 3 College of Horticulture, Sichuan Agricultural University, Chengdu, 611130 China

**Keywords:** Genome duplication, Plant breeding

## Abstract

Blood orange (BO) is a rare red-fleshed sweet orange (SWO) with a high anthocyanin content and is associated with numerous health-related benefits. Here, we reported a high-quality chromosome-scale genome assembly for Neixiu (NX) BO, reaching 336.63 Mb in length with contig and scaffold N50 values of 30.6 Mb. Furthermore, 96% of the assembled sequences were successfully anchored to 9 pseudo-chromosomes. The genome assembly also revealed the presence of 37.87% transposon elements and 7.64% tandem repeats, and the annotation of 30,395 protein-coding genes. A high level of genome synteny was observed between BO and SWO, further supporting their genetic similarity. The speciation event that gave rise to the *Citrus* species predated the duplication event found within them. The genome-wide variation between NX and SWO was also compared. This first high-quality BO genome will serve as a fundamental basis for future studies on functional genomics and genome evolution.

## Background & Summary

Sweet orange (SWO, *Citrus sinensis* L. Osbeck) is the most important citrus species^1^. SWO varieties are typically categorized into three subgroups based on their agronomical characteristics: common orange, navel orange, and blood orange (BO)^2^. BO stands out for brilliant red coloration of both flesh and rinds^3^, which is not usually found in *Citrus* L.^4,5^.

Anthocyanins, which belong to a large family of flavonoids, are accountable for the characteristic red color of BO^3^. In addition to contributing to pigmentation^3^, anthocyanins have various health-promoting benefits in humans, such as their antioxidant capacity and potential for cancer prevention^6^. As consumers become increasingly health-conscious, the popularity of BO has been growing worldwide^7^ because of its exceptional nutraceutical attributes, including vitamins, sugars, dietary fiber, minerals, and flavonoids, particularly anthocyanins^8^.

**Fig. 1:**
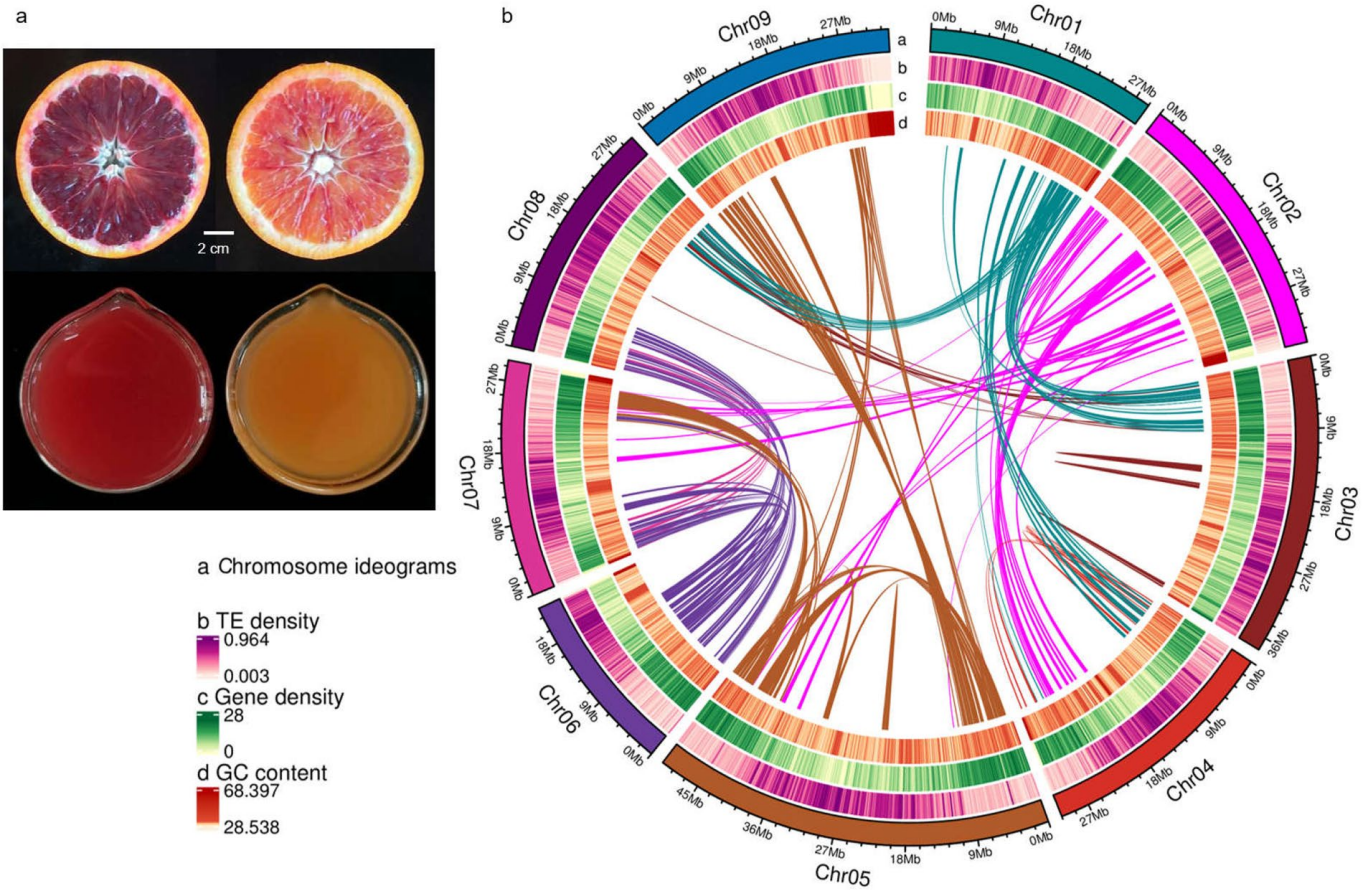
Morphological and genomic characteristics of Neixiu blood orange. (**a**) Fruit phenotypes of Neixiu (left) and Tarocco (right) blood oranges. (**b**) Genomic landscape of Neixiu blood orange, including chromosome ideogram, transposon element density, gene density, GC content, and intra-genome collinear blocks.

Moro, Tarocco, and Sanguinello are the three most important commercial BO types^9^. Moro has the deepest red color among the three varieties, followed by Sanguinello and Tarocco^4,9^. Tarocco is a medium-sized seedless variety famous for its peelability and sweetest taste^2^. In our long-term BO breeding program, we have discovered an unexpected and natural bud mutation of Tarocco, which we have named ‘内秀’ (Neixiu, NX). In Chinese wisdom, ‘内秀’ is used to describe a person who looks pretty ordinary, but he is intelligent in an understated way. Based on more than 5 years of careful observation, we found that NX surpasses common Tarocco in terms of both sweetness and redness in the Southwest region of China (Fig. 1). Consequently, we consider NX to be a highly promising BO cultivar.

Recent advancements in sequencing technology and associated bioinformatic tools have significantly expedited citrus genomic studies. To date, three genomes of the SWO variety have been released. In 2013, the first draft of a di-haploid SWO genome was complied using short Illumina reads^10^. Subsequently, Wang *et al*.^11^ successfully generated a *de novo* reference genome of the di-haploid SWO using the Nanopore ultra-long and PacBio long-read sequencing platforms. More recently, Wu *et al*. ^12^ accomplished the assembly of a diploid SWO genome at the chromosome level, specifically for the ‘Valencia’ variety. However, it is worth noting that genomic data for this important BO in the citrus industry is currently unavailable. In the investigation of BO functional genomics and genetics, the initial task involves the interpretation of genomic data.

Therefore, in the present study, we constructed a high-quality chromosome-scale genome assembly of BO by combining Illumina sequencing, third-generation circular consensus sequencing (CCS), and high-throughput chromosome conformation capture (Hi-C) sequencing. This integrated methodology resulted in a genome size of approximately 336.63 Mb, with a contig N50 value of 30.6 Mb. A total of 96% of the assembled sequences were successfully anchored to nine pseudo-chromosomes (Table 1). To investigate the evolutionary patterns of genes and genomes, comparative genomic analyses were performed on the BO genome and 11 other genomes representing various plant species. The study presents the first high-quality chromosome-scale genome of BO. The dataset generated from this research will significantly contribute to the advancement of our knowledge in BO functional genomics and the trajectory of citrus genomes.

**Table 1:** Assembly and assessment of Neixiu blood orange genome.

Parameter		Neixiu blood orange
Genome-sequencing depth (X)	Illumina sequencing	74.66
	PacBio sequencing	65
	Hi-C	165
PacBio*	Total contig length (Mb)	336.63
	Total contig No.	102
	Contig N50 (Mb)	35.13
	Contig N90 (Mb)	22.87
	Longest contig length (Mb)	40.3
	GC content (%)	37
Hi-C final genome assembly	Total contig length (Mb)	336.63
	Total contig No.	107
	Contig N50 (Mb)	30.6
	Contig N90 (Mb)	6.4
	Longest contig length (Mb)	50.16
	Total scaffold lengh (Mb)	336.63
	Total scaffold No.	106
	Scaffold N50 (Mb)	30.6
	Scaffold N90 (Mb)	6.4
	Longest scaffold length (Mb)	50.16
	GC content (%)	37
	% of sequence anchored on chromosome	96
CEGMA assessment	%of 458 CEGs present in assemblies	98.25
	% of 248 highly conserved CEGs present	95
BUSCO assessment	Complete BUSCOs	1585 (98.20%)
	Complete and single-copy BUSCOs	1519 (94.11%)
	Complete and duplicated BUSCOs	66 (4.09%)
	Fragmented BUSCOs	7 (0.43%)
	Missing BUSCOs	22 (1.36%)
	Total Lineage BUSCOs	1,614
Illuminal mapping	Mapped reads	158,405,429 (97.66%)
	Properly mapped reads	134,472,508 (82.91%)
HiFi long read mapping	Mapped reads	1,118,919 (99.58%)
	Properly mapped reads	0 (0%)
	Average sequencing depth	58
	Coverage ratio_1X (%)	99.96%
	Coverage ratio_5X (%)	99.5
	Coverage ratio_10X (%)	98.88
	Coverage ratio_20X (%)	95.94

## Methods

### Plant materials

For genome sequencing, young leaf samples were randomly collected from five-year-old NX trees. Samples were immediately frozen in liquid nitrogen, followed by preservation at −80 °C until DNA and RNA extraction. For RNA extraction, fresh plant tissues including leaves, fruits, buds, roots, and branches were obtained from the same tree. The ‘Valencia’ SWO^11^ was used in the bioinformatics analysis.

### Library construction and sequencing

Genomic DNA and total RNA were extracted using DNeasy Plant Mini Kit and RNeasy Plus Mini Kit (Qiagen, Valencia, CA, USA), respectively, according to the manufacturer’s instructions. After extraction, short-read (350-bp) libraries were constructed using a library construction kit (Illumina, San Diego, CA, USA) and then sequenced on a Novaseq 6000 platform (Illumina), which finally generated a total of 24.21 Gb of raw data, covering 74.66 × of the genome. The resulting clean reads were used for genome surveys, including the evaluation of genome size, GC content, and heterozygosity.

PacBio sequencing libraries were constructed by Biomarker Technologies Corporation (Beijing, China) using the SMRTbell® express template prep kit 2.0 (PacBio, Menlo Park, CA, USA). Before library preparation, genomic DNA was sheared into 15 kb fragments using Megaruptor® 3 (Diagenode, Denville, NJ, USA). A total of 21.21 Gb high-fidelity (HiFi) clean data with an N50 value of 19.36 kb and an average read length of 18.88 kb were produced using the CCS mode on a PacBio Sequel II platform with the Sequel sequencing kit 2.0 (PacBio). These data are equivalent to 65 × coverage of the entire genome.

Hi-C libraries with 300~700-bp insert size were prepared following Rao *et al*.^13^ and sequenced on a NovaSeq 6000 platform (Illumina). This sequencing generated approximately 55.6548 Gb reads.

### Genome survey and assembly

**Fig. 2:**
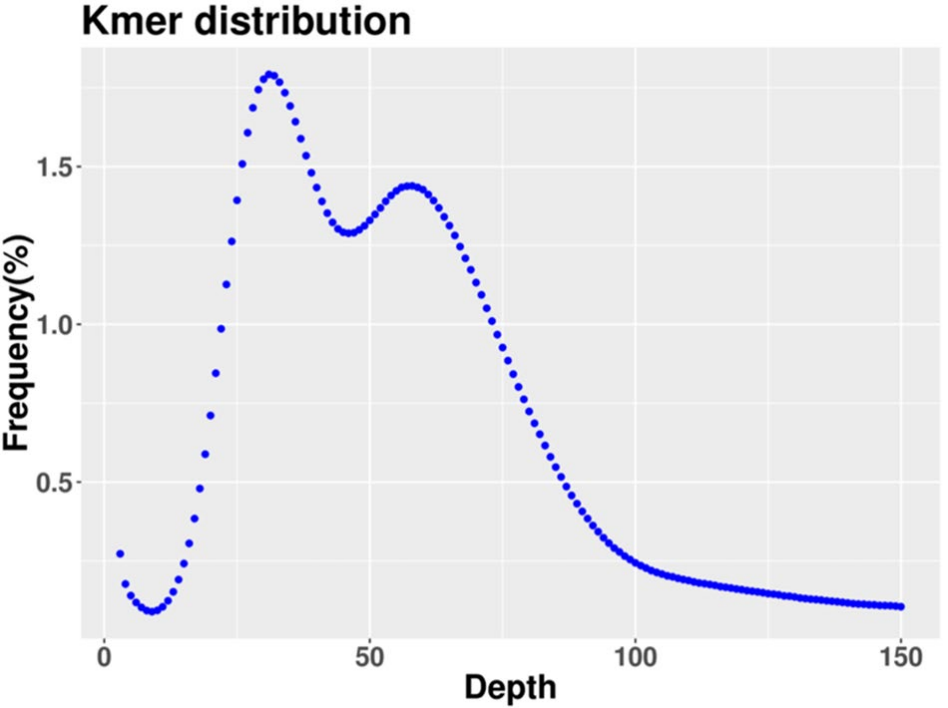
Frequency distribution of the 19-mer analysis. The x-axis represented the K-mer depth and y-axis represented the frequency of K-mer correspond to the depth.

Illumina short reads were filtered using fastp^14^ to remove low-quality reads and adapters before genome size estimation. SOAP v.2.21^15^ was used for the initial assembly. The frequencies of 19 K-mers were determined using Jellyfish v.2.1.4^16^. Based on these analysis, the genome size was estimated to be 324.21 Mb, with a heterozygosity rate of 1.82%, a repeat element ratio of 43.81%, and a GC content of 35.63% (Fig. 2).

The HiFi long reads were subjected to genome assembly using Hifiasm v.0.16^17^, resulting in a contig length of 494.34 Mb and a contig N50 value of 30.18 Mb. Redundant contigs caused by heterozygosity were removed using Purge_dups^18^, resulting in a contig length of 336.63 Mb and a contig N50 value of 35.13 Mb (Table 1).

**Fig. 3:**
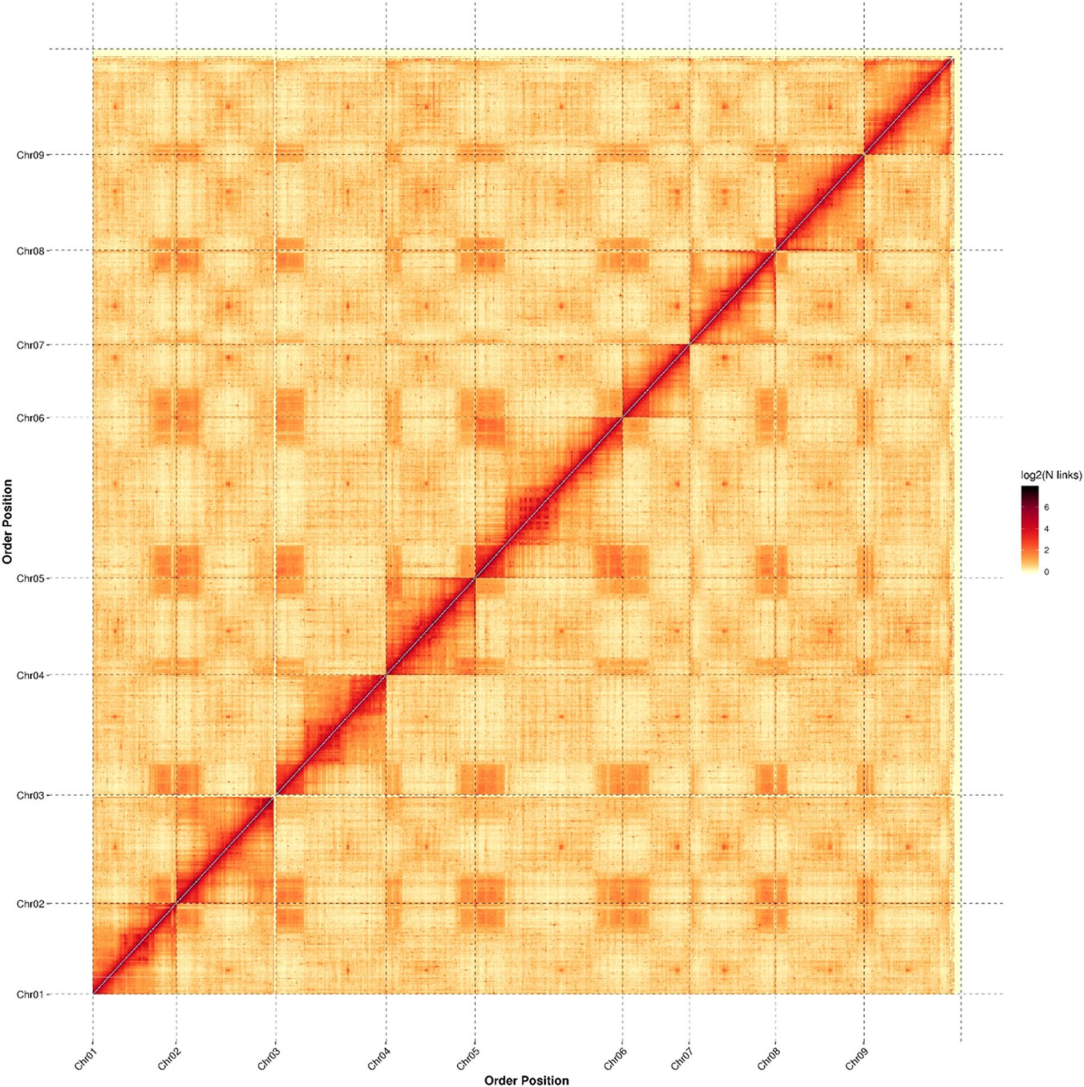
Hi-C interaction heatmap for Neixiu blood orange. The map shows scaffolded and independently assembled chromsomes at high resolution.

Adaptors and low-quality Hi-C reads were filtered using HiC-Pro v.2.10.0^19^, retaining only uniquely mapped paired-end reads with a mapping quality greater than 20. The scaffolds/contigs underwent clustering, ordering, and orientation onto chromosomes using LACHESIS^20^. Subsequently, any placement or orientation errors that displayed distinct chromatin interaction patterns were manually adjusted. These scaffolds were anchored to nine pseudo-chromosomes, which accounted for 96% of the assembled genome (Fig. 3). The Hi-C scaffolding process ultimately achieved the final chromosome-scale genome assembly of BO (336.63 Mb) with contig and scaffold N50 values of 30.6 Mb (Table 1).

### Repeat element identification

Transposon elements (TEs) were identified by combining *de novo* and homology-based strategies using RepeatModeler2 v.2.0.4^21^. This involved in the automated execution of two repeat-finding programs (RECON v.1.0.8 and RepeatScout v.1.0.6) and the classification of prediction results using RepeatClassifier^21^, which entailed a search of Dfam v.3.5^22^. LTRharvest v.1.06^23^ and LTR_finder v.1.5.10^24^ were used identify the full-length repeat retrotransposons (LTR-RTs). High-quality intact full-length LTR-RTs and non-redundant LTR libraries were produced from the outputs of LTR_retriever v.2.9.0^25^. By combining the *de novo* TE library with known TEs in RepBase v.19.06^26^, REXdb v.3.0^27^, and Dfam v.3.5^22^, a non-redundant species-specific TE library was obtained. The final TEs were identified and classified through a homology search against the library using RepeatMasker v.4.1.4^28^. Tandem repeats were annotated using Tandem Repeats Finder^29^ and MISA v.2.1^30^. In the BO genome, we identified 127.82 Mb (37.97%) of TEs and 25.72 Mb (7.64%) of tandem repeats. The majority of repeats (28.06%) were Class I retrotransposons, dominated by gypsy (13.04%) and copia (7.52%) elements. Class II DNA transposons accounted for 9.91% of the BO genome (Table 2).

**Table 2:** Repetitive elements and their proportions in Neixiu blood orange.

Repeat elements	Number	Length (bp)	Proportion in genome (%)
ClassI:Retroelement	123,086	94,456,213	28.06
ClassI/DIRS	1	39	0
ClassI/LINE	20,394	6,332,042	1.88
ClassI/LTR/Caulimovirus	4,520	6,355,837	1.89
ClassI/LTR/Copia	22,843	25,298,113	7.52
ClassI/LTR/ERV	1,461	95,938	0.03
ClassI/LTR/Gypsy	34,245	43,892,681	13.04
ClassI/LTR/Ngaro	327	60,967	0.02
ClassI/LTR/Pao	109	19,291	0.01
ClassI/LTR/Unknown	34,672	11,679,287	3.47
ClassI/SINE	4,514	722,018	0.21
ClassII:DNA transposon	97,837	33,366,886	9.91
ClassII/CACTA	1,981	1,036,105	0.31
ClassII/Crypton	28	1,108	0
ClassII/Dada	185	9,856	0
ClassII/Ginger	40	2,276	0
ClassII/Helitron	1,022	637,415	0.19
ClassII/IS3EU	143	8,085	0
ClassII/Kolobok	185	11,724	0
ClassII/Maverick	106	6,780	0
ClassII/Merlin	145	6,586	0
ClassII/Mutator	3,570	2,679,529	0.8
ClassII/P	78	4,843	0
ClassII/PIF-Harbinger	1,048	239,771	0.07
ClassII/PiggyBac	42	1,892	0
ClassII/Tc1-Mariner	379	57,572	0.02
ClassII/Unknown	83,531	26,958,741	8.01
ClassII/Zisupton	356	56,794	0.02
ClassII/hAT	4,998	1,647,809	0.49
Unknown	17	1,263	0
Transposable elements	220,940	#########	37.97
microsatellite(1–9 bp units)	181,162	2,896,325	0.86
minisatellite(10–99 bp units)	58,599	5,196,820	1.54
satellite(> = 100 bp units)	7,805	17,625,606	5.24
Tandem repeats	247,566	25,718,751	7.64

### Protein-coding genes prediction

A total of 30,395 protein-coding genes have been annotated by incorporating *de novo*, homology, and transcript-based predictions (Table 3). The *de novo* gene models were predicted using Augustus v.3.2.2^31^ and SNAP v.2006-07-28^32^. GeMoMa v.1.7^33^ was used for homology-based predictions by annotating the gene models in BO with amino acid sequences from *C. grandis*, SWO, *Poncirus trifoliata*, and *Arabidopsis thaliana* genomes. For transcript-based prediction, RNA-seq data was mapped to the reference genome using HISAT v.2.2.1^34^ and quantified with StringTie v.2.1.4^35^. Genes were predicted from the assembled transcripts using GeneMarkS-T v.5.1^36^. Another transcript-based prediction method was performed using Trinity v.2.1.1^37^. Program to Assemble Spliced Alignments (PASA) v.2.4.1^38^ was used to predict gene models based on the unigenes. The genes predicted in the aforementioned three annotation files were merged using EVidenceModeler v.1.1.1^39^, and the final gene set was updated using PASA v.2.4.1^38^. Each gene exhibited an average of 5.02 exons, with a mean gene length of 3489.94 bp and a coding sequence size of 1152.21 bp. The average lengths of exons and intros were 1440.51 and 2049.43 bp, respectively (Table 3).

**Table 3:** Genome annotation of Neixiu blood orange.

Annotation	Type	Neixiu blood orange
Gene prediciton	Gene number	30,395
	Gene length (bp)	106,076,691
	Average gene length (bp)	3489.94
	Exon length (bp)	43,784,235
	Average exon length (bp)	1440.51
	Exon number	152,686
	Average exon number	5.02
	CDS length (bp)	35,021,391
	CDS number	1152.21
	Average CDS length (bp)	148,644
	Average CDS number per gene	4.89
	Intron length (bp)	62,292,456
	Average intron length (bp)	2049.43
	Intron number	122,291
	Averrage intron number per gene	4.02
Non-coding genes	rRNA number	5,339
	tRNA number	475
	miRNA number	162
	snRNA number	905
	snoRNA number	1,367
Gene function annotation	GO annotation	22,068 (72.6%)
	KEGG annotation	19,388 (63.79%)
	KOG annotation	13,861 (45.6%)
	Pfam annotation	21,797 (71.71%)
	Swissprot annotation	20,730 (68.2%)
	TrEMBL annotation	26,989 (88.79%)
	eggNOG annotation	21,881 (71.99%)
	Nr annotation	26,616 (87.57%)
	All annotated	27,223 (89.56%)
Motif annotation	Motif	1,068
	Domain	26,539

### Gene function annotation

To ascertain the functional characteristics, the predicted genes underwent annotation by aligning them with the gene ontology (GO), Kyoto Encyclopedia of Genes and Genomes (KEGG), eukaryotic orthologous groups (KOG), protein families (Pfam), SwissProt, TrEMBL, evolutionary genealogy of genes, non-supervised orthologous groups (eggNOG), and NCBI non-redundant protein (Nr) databases. Additionally, the motifs and domains were annotated using InterProscan v.5.27.66^40^. Based on the aforementioned multiple databases, a total of27,223 genes, accounting for 89.56% of the predicted protein-coding genes, were successfully annotated. Specifically, the GO, KEGG, KOG, Pfam, SwissProt, TrEMBL, Eggnog, and Nr databases annotated approximately 72.6%, 63.79%, 45.6%, 71.71%, 68.2%, 88.79%, 71.99%, and 87.57% of genes, respectively (Table 3).

### Non-coding RNA annotation

Transfer RNA (tRNA) and ribosomal RNA (rRNA) were identified using tRNAscan-SE v.1.3.1^41^ and Barmap v.0.9.0^42^, respectively. Furthermore, other non-coding RNAs (ncRNAs), including microRNA (miRNA), small nucleolar RNA (snoRNA), and small nuclear RNA (snRNA), were identified using Infernal v.1.1.2^43^ by searching against Rfam v.14.1^44^. In total, 8,248 ncRNAs (5,339 rRNAs, 475 tRNAs, 162 miRNAs, 905 snRNAs, and 1,367 snoRNAs) were identified in the BO genome (Table 3).

### Comparative genomics analysis

An all-against-all protein sequence similarity search was conducted between the BO genome and 11 representative plant species (*P. trifoliata*, *Malus domestica*, *Arabidopsis thaliana*, *Solanum lycopersicum*, *C. sinensis*, *Oryza sativa*, *Ziziphus jujuba*, *C. clementina*, *Amborella trichopoda*, *Vitis vinifera*, and *C. unshiu*) using Orthofinder v.2.3.8^45^ with the diamond alignment method. The resulting gene families were then annotated using Panther v.15^46^. Unique gene families in BO were subjected to GO and KEGG enrichment analysis using ClusterProfiler v.3.14.0^47^.

**Fig. 4:**
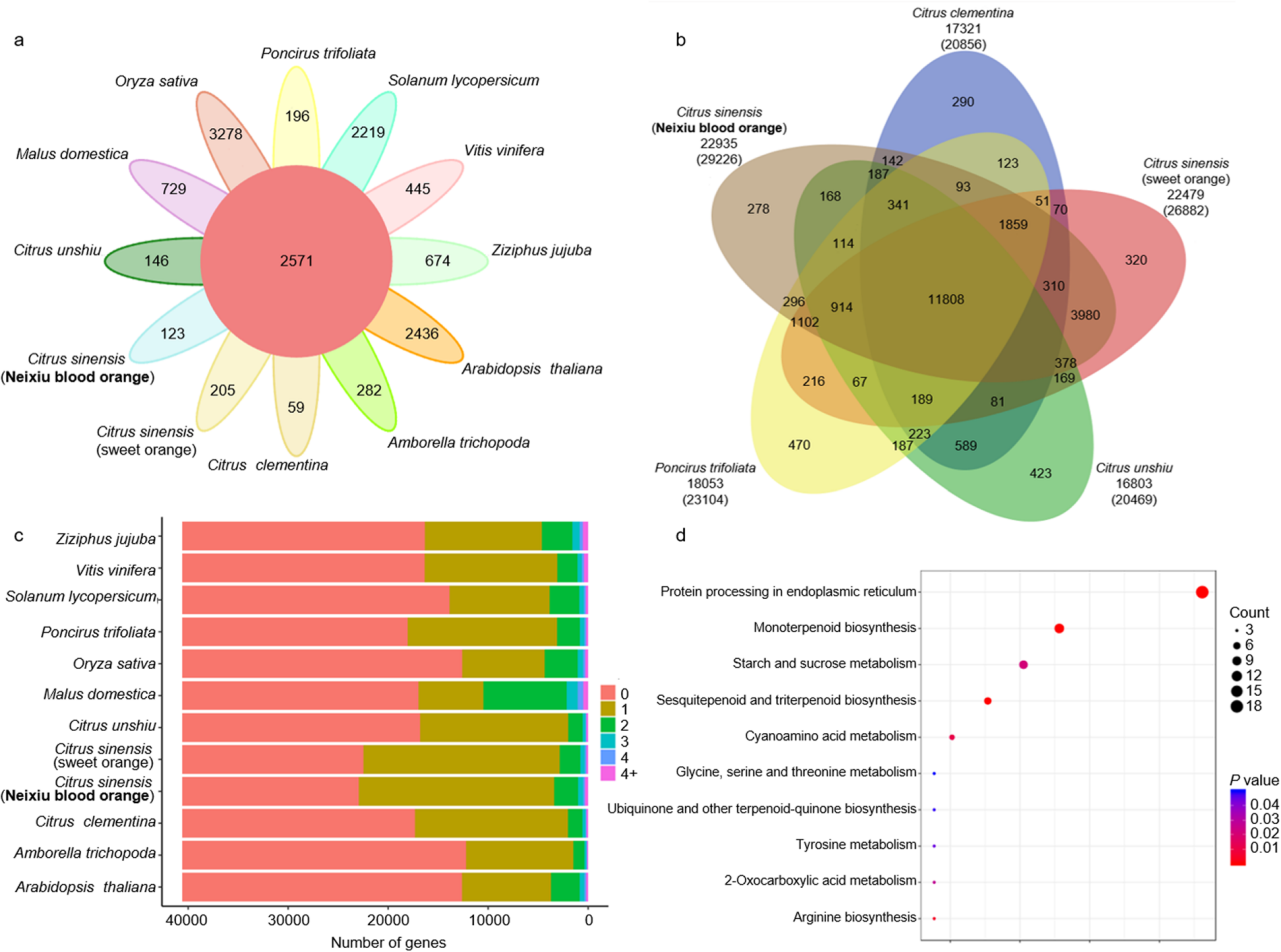
Comparative genomic analysis of Neixiu blood orange and other 11 representative plant species. (**a**) Gene family cluster petal map of Neixiu blood orange and other 11 representative plant species. The central circle represents common gene families, and the outer petals represent specific gene families. (**b**) Venn diagram showing gene family clusters of five Rutaceae species. (**c**) The number of gene copies and their distribution among 12 plant species. (**d**) KEGG enrichment analysis of genes specific to Neixiu blood orange.

A total of 40,592 gene families were identified, including 2,571 gene families that shared among these species and 123 that were specific to BO (Fig. 4a). Notably, a significant proportion of the genes in BO and the other 11 species were found to be single-copy genes (Fig. 4b). Among the Rutaceae species, including BO, *C. sinensis*, *C. clementina*, *C. unshiu*, and *P. trifoliata*, a total of 11,808 gene families were shared with 278 gene families specific to BO (Fig. 4c). Further KEGG analysis revealed that these BO specific genes were significantly enriched in various pathways, such as protein processing in the endoplasmic reticulum, monoterpenoid biosynthesis, and starch and sucrose metabolism (Fig. 4d).

### Phylogenetic and evolutional analyses

**Fig. 5:**
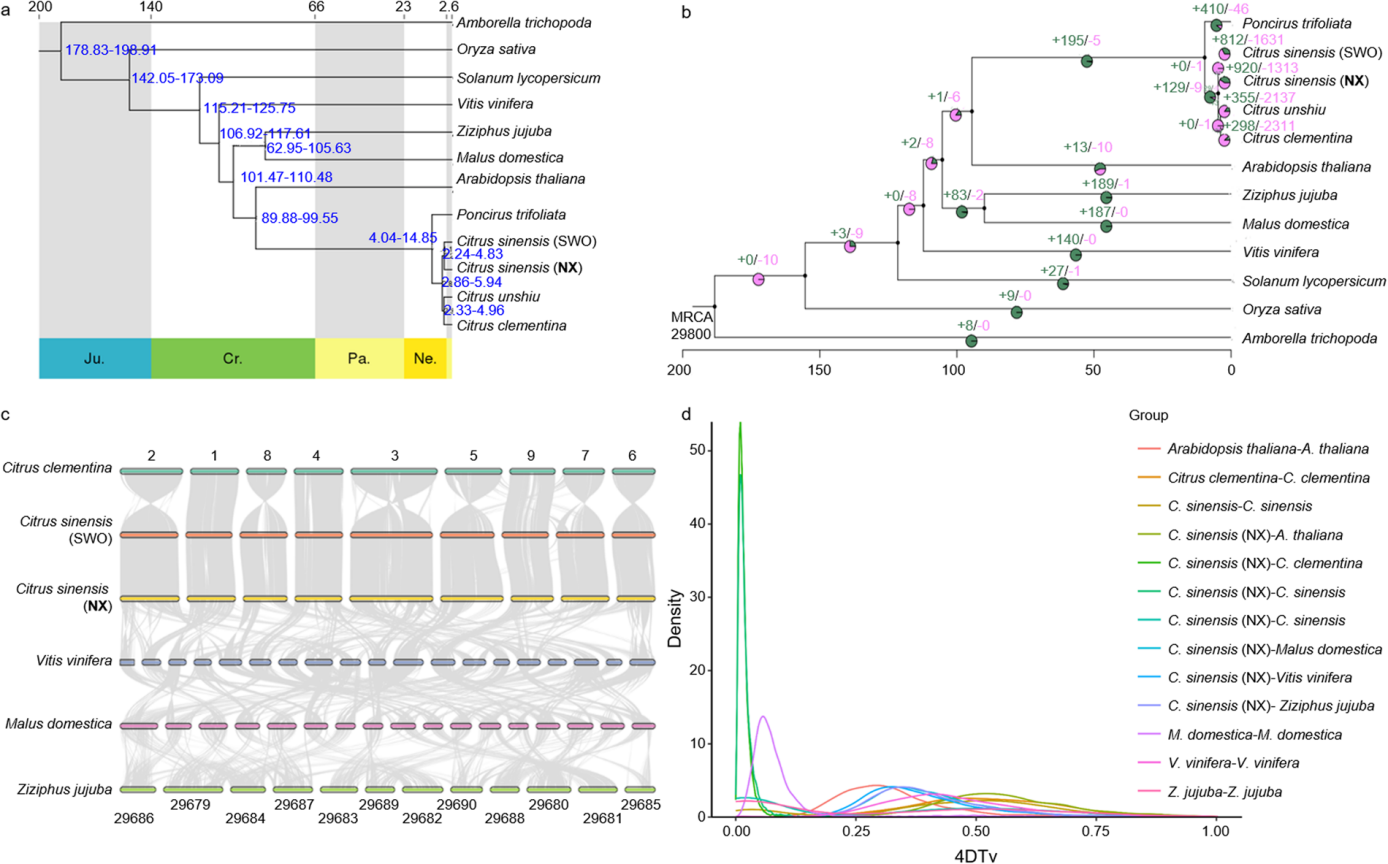
Evolution analyses of Neixiu blood orange and other 11 representative plant species. (**a**) Phylogenetic tree showing the relationships among 12 species with divergence time. The top and bottom of the tree represent the absolute age (millions of years) and geological time (Neogence, Ne.; Paleogence, Pa.; Cretaceous, Cr.; and Jurassic, Ju.). All the nodes have 100% boostrap support. (**b**) Phylogenetic tree showing the relationships among 12 species with gene family expansion (green color) and contraction (pink color). MRCA, most recent common ancestor. (**c**) Genome synteny among Neixiu blood orange, sweet orange, *Citrus clementina*, *Vitis vinifera*, and *Malus domestica*. (**d**) Distribution of the 4DTv rates among the paralogs of the studied species.

A phylogenetic tree was constructed using IQ-Tree^48^ based on 594 single-copy gene sequences obtained from these 12 species. The alignment of orthologous gene sequence was performed independently using MAFFT v.7.490^49^, followed by the conversion of protein alignments to nucleotide sequence alignments using PAL2NAL v.14^50^. The alignments were then refined using the Gblocks 0.91b^51^. Clean super-alignments were used to construct a maximum likelihood phylogenetic tree using IQ-Tree^48^ with a fitted model of GTR + F + I + G4 suggested by ModelFinder^52^. The resulting tree revealed BO is a sister clade to *C. sinensis*, indicating a closer relationship with SWO than with mandarins (*C. unshiu* and *C. clementina*) (Fig. 5a).

The divergence time among the 12 plant species was calculated using MCMCTree in the PAML v.4.9^53^ with 95% confidence intervals. TimeTree^54^ calibration points were used to infer the divergence time. The calculated divergence times were as follows: *C. sinensis*-*Amborella trichopoda*, 179.0–199.1 million years ago (mya); *C. sinensis*-*C. clementina*, 1.5–5.7 mya; *C. sinensis*-*O. sativa*, 143.0–174.8 mya; *C. sinensis*-*S. lycopersicum*, 112.4–125.0 mya; *C. sinensis*-*M. domestica*, 102.0–113.8 mya; and *C. sinensis*-*Arabidopsis thaliana*, 90.0–99.9 mya. These estimates were subsequently used to correct the fossil time obtained from the software algorithm. *Amborella trichopoda* was used as the outgroup for conducting maximum-likelihood-based phylogenetic analyses. The divergence time between the SWO and BO (2.24–4.83 mya) was comparatively more recent compared than that of *C. unshiu* and *C. clementina* (2.33–4.96 mya), while the divergence time of oranges and mandarins (2.98–5.94 mya) was found to be the earliest among the four *Citrus* species (Fig. 5a). The gene expansion and contraction of the gene families were determined using Computation Analysis of gene Family Evolution (CAFE)^55^ v.3.1. In total, 920 and 1,313 gene families expanded and contracted in the BO genome, respectively (Fig. 5b).

### Synteny and whole-genome duplication (WGD) analysis

To better understand the evolutionary history of BO, we performed a genomic collinearity analysis of BO, SWO, *C. clementina*, *V. vinifera*, *M. domestica*, and *Z. jujube*. Homologous gene pairs were identified through a comparison of the genomic sequences of two species using the DIAMOND v.0.9.29.130^56^. Subsequently, JCVI v.0.9.13 was used to visualize collinear blocks identified using homologous gene pairs in MCScanX^57^. A significant level of synteny was observed between the genomes of BO and SWO. The BO chromosomes were mapped with more fragments in the SWO than in *C. clementina* (Fig. 5c).

To determine the occurrence of WGD events, a combination of the synonymous mutation rate (Ks) and fourfold synonymous third-codon transversion (4DTv) was employed. This analysis was conducted using WGD v.1.1.1^58^ and a publicly available script (https://github.com/JinfengChen/Scripts). The 4DTv values of BO, SWO, and *C. clementina* reached a peak of 0.5, indicating the occurrence of WGD events in *Citrus*. The *Citrus* speciation event took place prior to the duplication event observed in *Citrus* species, evidenced by the pairwise 4DTv distribution of BO compared to *M. domestica, V. vinifera*, *Z. jujuba*, and *Arabidopsis thaliana* (Fig. 5d).

### Genome-wide variation analysis

To investigate the genomic differences between BO and SWO, we used the assembled NX as the reference genome and the most recent chromosome-level phased diploid Valencia SWO genome, as published by Wu *et al*.^12^, for conducting genome-wide alignments with the nucmer, delter-filter, and show-coord programs from MUMmer v.4.0^59^. This analysis yielded a total of 1,275,362 single-nucleotide polymorphism (SNP) differences and 295,024 insertion-deletions (InDels), including 170,365 insertions and 124,659 deletions. Subsequently, the filtered delta files were subjected to SyRI^60^ for the identification of structural variations (SVs) in. A total of 694 copy number variations (CNVs) were found in SWO genome compared to the BO genome, with 362 copies increased and 332 copies lost in number in the SWO genome. Presence-absence variations (PAVs) are major contributors to genome structural variations, impacting both phenotypic and genomic variability^61^. We detected 1,081 present and 1,340 absent variations. GO and KEGG enrichment analyses were conducted using clusterProfiler v.3.14.10^47^ for genes where mutations were detected. ANNOVAR^62^ was used for the functional annotation of genetic variants.

## Data Records

The genome sequences, PacBio raw data, and Hic-C raw data have been deposited to the NCBI SRA database^63,64^ and the genome gff annotation file was uploaded to^65^. Genome estimation, statistics of assembled genome sequences, integrated function annotation, statistics of gene family clustering, and list of the expanded and constracted gene families were submitted at the Figshare^66^.

## Technical Validation

The assessment of the final assembled genome completeness and quality involved the implementation of (1) Core Eukaryotic Genes Mapping Approach (CEGMA) v. 2.5^67^, (2) Benchmarking Universal Single-Copy Orthologs (BUSCO) v. 5.2.1^68^, (3) alignment using Burrows–Wheeler Aligner (BWA)^69^ with Illumina data, and (4) alignment using Minimap 2^70^ with HiFi reads.

The evaluation of the final assembled genome’s integrity was performed by referencing the CEGMA database, which contains 458 core eukaryotic genes (CEGs) and 248 highly conserved CEGs, and by employing tblastn, genewise, and geneid software^67^. The assembled genome contained 98.25% (450) of CEGs and 95.16% (236) of highly conserved CEGs, suggesting that it contained most CEGs. To evaluate the integrity of the assembly, BUSCO^68^ analysis was conducted using the Embryophyta database OrthoDB v. 10 (http://cegg.unige.ch/orthodb), which encompasses 1,614 orthologous single-copy genes. The assembled genome contained 1,585 (98.20%) of these genes. Mapping of Illumina short reads and HiFi long reads to the assembled genome revealed that approximately 97.66% and 99.58% of the reads, respectively, aligned successfully (Table 1).

To ensure the reliability of the MCMCTree analyses, the correlated molecular clock and JC69 model were employed, and all relevant computations were performed twice to ensure consistency. The correlation between two iterations in this test is 1.

In order to evaluate the reliability of the inference in constructing the phylogenetic tree, 1000 bootstrap replicates were performed for each branch.

## Data Availability

Fastp: -q 10 -u 50 -y -g -Y 10 -e 20 -l 100 -b 150 -B 150 SOAP: -m 260 -x 440 Jellyfish: -h 100000 Hifiasm: l = 2, n = 3 LACHESIS: CLUSTER_MIN_RE_SITES = 31;CLUSTER_MAX_LINK_DENSITY = 2;ORDER_MIN_N_RES_IN_TRUNK = 15;ORDER_MIN_N_RES_IN_SHREDS = 15 LTRharvest: -minlenltr 100 -maxlenltr 40000 -mintsd 4 -maxtsd 6 -motif TGCA -motifmis 1 -similar 85 -vic 10 -seed 20 -seqids yes LTR_finder: -D 40000 -d 100 -L 9000 -l 50 -p 20 -C -M 0.9 Diamond alignment (Orthofinder): e ≤ 1e^−3^ MAFFFT: --localpair --maxiterate 1000 Gblocks: -b5 = h PAML: burnin 5000000; sampfreq. 30; nsample 10000000 DIAMOND v. 0.9.29.13: e < 1e^−5^, C > 0.5 MCScanX: -m 15 Nucmer program from MUMmer v. 4.0: --maxmatch -c 500 -b 500 -l 100 -t 6 Delta-filter program from MUMmer v. 4.0: -1 -i 90 -l 500 Show-coords program from MUMmer v. 4.0: -THrd
